# Pridopidine Induces Functional Neurorestoration Via the Sigma-1 Receptor in a Mouse Model of Parkinson’s Disease

**DOI:** 10.1007/s13311-018-00699-9

**Published:** 2019-02-12

**Authors:** Veronica Francardo, Michal Geva, Francesco Bez, Quentin Denis, Lilach Steiner, Michael R. Hayden, M. Angela Cenci

**Affiliations:** 1 Basal Ganglia Pathophysiology Unit, Department of Experimental Medical Science, Lund University, BMC F11, Lund, Sweden; 2 Prilenia Therapeutics, Herzliya, Israel; 3 Teva Pharmaceutical Industries Global Research and Development, Netanya, Israel

**Keywords:** MAM, endoplasmic reticulum, disease modification, neuroprotection, neuroinflammation, plasticity

## Abstract

Pridopidine is a small molecule in clinical development for the treatment of Huntington’s disease. It was recently found to have high binding affinity to the sigma-1 receptor, a chaperone protein involved in cellular defense mechanisms and neuroplasticity. Here, we have evaluated the neuroprotective and neurorestorative effects of pridopidine in a unilateral 6-hydroxydopamine (6-OHDA) lesion model of parkinsonism in mice. By 5 weeks of daily administration, a low dose of pridopidine (0.3 mg/kg) had significantly improved deficits in forelimb use (cylinder test, stepping test) and abolished the ipsilateral rotational bias typical of hemiparkinsonian animals. A higher dose of pridopidine (1 mg/kg) significantly improved only the rotational bias, with a trend towards improvement in forelimb use. The behavioral recovery induced by pridopidine 0.3 mg/kg was accompanied by a significant protection of nigral dopamine cell bodies, an increased dopaminergic fiber density in the striatum, and striatal upregulation of GDNF, BDNF, and phosphorylated ERK1/2. The beneficial effects of pridopidine 0.3 mg/kg were absent in 6-OHDA-lesioned mice lacking the sigma-1 receptor. Pharmacokinetic data confirmed that the effective dose of pridopidine reached brain concentrations sufficient to bind S1R. Our results are the first to show that pridopidine promotes functional neurorestoration in the damaged nigrostriatal system acting via the sigma-1 receptor.

**Electronic supplementary material:**

The online version of this article (10.1007/s13311-018-00699-9) contains supplementary material, which is available to authorized users.

## Introduction

The sigma-1 receptor (S1R) is a ligand-operated chaperone protein, enriched at mitochondria-associated endoplasmic reticulum membranes (MAMs), supporting several pathways of cell defense and survival [1]. Neuroprotective and/or neurorestorative properties of S1R agonists have been reported in several models of neurodegenerative disorders (in particular, Alzheimer’s disease and amyotrophic lateral sclerosis) or acquired brain injury [2–10]. These beneficial effects have been attributed to multiple mechanisms, including upregulation of anti-apoptotic genes, reduced microglial activation, reduced generation of reactive oxygen species (ROS) and nitric oxide (NO), and an increased secretion of trophic factors. Moreover, the S1R has recently been found to confer protection of nigrostriatal dopamine neurons against age- and alpha-synuclein-dependent degeneration [11].

Parkinson’s disease (PD) is an age-related neurodegenerative disease characterized by a severe loss of nigrostriatal dopamine neurons, which is attributed to reciprocal interactions between alpha-synuclein aggregation, oxidative stress, mitochondrial dysfunction, and neuroinflammation [12–16]. In theory, all factors involved in this pathogenic network could be attacked by increasing the biological activities of S1R.

We have previously evaluated the selective S1R agonist PRE-084 in mice with 6-OHDA-induced nigrostriatal degeneration and found that daily administration of PRE-084 at the dose of 0.3 mg/kg produced significant motor recovery by 5 weeks, accompanied by a partial protection of nigral dopaminergic cell bodies, an increase in striatal dopaminergic fiber density, an upregulation of trophic factor levels, and a reduced number of activated microglial cells [17].

In the present study, we set out to evaluate the effects of pridopidine as a potential S1R agonist in the same animal model of parkinsonism. Pridopidine (ACR 16) is an experimental drug candidate originally developed by Arvid Carlsson Research laboratories to stabilize states of altered dopamine transmission [18]. Accordingly, it was reported to normalize either hyper- or hypolocomotion in rats depending on the prevailing dopaminergic tone in the brain [19]. Pridopidine has been found to improve motor function in Huntington’s disease (HD) patients and animal models of HD [20–24]. Although its motor effects were originally attributed to low-affinity antagonism of dopamine D2 receptors (D2Rs), *in vivo* PET studies show that behaviorally relevant doses of pridopidine are more likely to occupy the S1R than the D2R because of a much larger binding affinity at the former compared to the latter target (Ki for rat S1R 69.7 nM [25]; IC50 and Ki for D2R ~ 10 μM [26, 27]).

Our results show that, at a low dose, pridopidine produces a functional neurorestoration of the damaged nigrostriatal pathway, accompanied by reduced microglia activation and upregulation of neurotrophic factors in the striatum. Pridopidine treatment did not show any of these beneficial effects in 6-OHDA-lesioned mice lacking S1R. Pharmacokinetic data confirmed that pridopidine, at the effective dosage, was present in the brain at a concentration sufficient to bind S1Rs. These results are the first to demonstrate that, by acting like a S1R agonist, pridopidine can both protect degenerating dopamine neurons and reinstate a functionally significant dopaminergic innervation in the motor striatum.

## Methods

### Animals

The study was performed in C57Bl6J mice (Charles River Laboratories, Sulzfeld, Baden-Württemberg, Germany) weighing approx. 25 g and having an age of 8 to 9 weeks at the beginning of the experiments. A total of 65 male wild-type mice and 52 S1R knockout (KO) mice of both genders were used. S1R knockout mice were bred on a C57BL6J background at Lund University. Our S1R KO line is derived from the well-characterized Sigmar1^Gt(OST422756)Lex^ mouse strain distributed by the Mutant Mouse Resource Regional Centre (MMRRC) at the University of California, Davis (CA). The mice were housed under a 12-h light/dark cycle with free access to food and water. Housing conditions and experimental treatments had been approved by the Malmö-Lund Ethical Committee on Animal Research.

### 6-OHDA Lesions

Lesions were performed according to previously described procedures [17]. Briefly, mice were anesthetized with isoflurane (Isoba®vet, Apoteksbolaget, Solna, Stockholm, Sweden) and placed in a stereotaxic frame on a flat-skull position. 6-OHDA-HCl (Sigma-Aldrich AB, Stockholm, Sweden) was freshly dissolved in 0.02% ascorbate-saline at the concentration of 3.2 mg free base per milliliter. One microliter of toxin solution per site was injected into the right striatum at the following coordinates (given in mm relative to the bregma, sagittal suture, and dural surface, cf. Paxinos and Franklin, 2001): AP + 1.0, ML − 2.1, and DV − 2.9, site 1, and AP + 0.3, ML − 2.3, and DV − 2.9, site 2. The solution was injected via a glass capillary (tip diameter ~ 50 μm) at the rate of 0.5 μL/min, and the capillary was left in place for 2 min after each injection.

### Treatments

Pridopidine was dissolved in physiological saline immediately before use and injected at the volume of 0.1 mL/10 g body weight in a single subcutaneous (s.c.) injection per day. The first injection was given immediately upon completion of the 6-OHDA infusions. In the first experiment, pridopidine was administered at either 0.3 or 1.0 mg/kg. These doses were chosen based on our previous study with the selective sigma-1 agonist PRE-084 [17], which has a nanomolar binding affinity for S1R similar to that of pridopidine. After establishing that only 0.3 mg/kg pridopidine produced a functionally relevant neurorestoration, this dose was selected for the rest of the study. Pridopidine was always administered at about 2 p.m., while behavioral tests were always performed in the morning between 9:00 and 10:30 a.m. (this time lag was allowed in order to rule out confounders due to potential motor effects of pridopidine).

### Behavioral Tests

All the mice participating in this study underwent 3 behavioral tests once a week.

i) *Spontaneous rotational activity*

Postural and locomotor asymmetries resulting from unilateral nigrostriatal denervation were assessed using a test of spontaneous rotation in an open field. In this test, animals transiently turn towards the side ipsilateral to the 6-OHDA lesion in response to the novelty of the test environment, and the turning response gradually declines in all animals upon test repetition [17, 28].

Briefly, each mouse was placed individually in the center of the open field (50 × 50 cm) and immediately video-recorded for 10 min, corresponding to the period of maximal activity [28]. Full rotations (continuous turns of 360°) were counted off-line by an experimentally blinded investigator. Data are expressed as the total number of net full turns ipsilateral to the lesion per test session.

ii) *Cylinder test*

Forelimb use asymmetry during vertical exploration provides a validated measure of forelimb akinesia in hemiparkinsonian rodents [29]. The test was executed as in [17]. Briefly, mice were placed individually in a glass cylinder (10 cm diameter, 14 cm height) and videorecorded for 3 to 5 min. The number of supporting wall contacts performed independently by the paw contralateral to the lesion was expressed as a percentage of all supporting wall contacts in each session.

iii) *Stepping test*

An impaired capacity to perform adjusting steps during experimenter-imposed movements (stepping test) reflects both akinetic and postural deficits relevant to PD [30, 31]. This test was performed as in [17]. The mouse was placed at the entrance of a custom-made plastic corridor (7 cm wide, 1 m long, flanked by 10-cm-high walls), gently lifted by the tail, and pulled backwards with a fixed speed (1 m/4 s). Each trial was video-recorded, and the footage was used to count the number of adjusting steps performed by each forelimb. Trials in which mice turned their body by 90° towards the walls of the corridor (in an attempt to escape or explore) were discarded. In each session, mice were tested until 3 valid trials per animal were obtained. Results were expressed as the percentage of steps performed by the forelimb contralateral to the lesion (left) over the total number of steps (mean of 3 trials per session).

### Brain Preparation

Mice allocated to immunohistochemistry were perfusion-fixed on the day after the last drug/saline administration (interval of approx. 20 h from the last injection). Animals were deeply anesthetized with sodium pentobarbital (240 mg/kg, i.p.) and transcardially perfused with 0.9% saline solution, followed by ice-cold buffered 4% paraformaldehyde (pH 7.4). After rapid extraction, brains were immersed in the same fixative for 2 h and then cryoprotected in ice-cold phosphate-buffered 25% sucrose solution overnight. Coronal sections of 30 μm thickness were cut on a sliding microtome and stored at − 20 °C in a nonfreezing solution (30% ethylene glycol and 30% glycerol in 0.1 M phosphate buffer).

Animals prepared for Western blot analysis were euthanized by cervical dislocation; their brains were rapidly extracted and frozen on crushed dry ice. Tissue samples were dissected out in a cryostat chamber (− 14 °C). A coronal brain slice spanning across rostrocaudal levels, + 1.18 to − 0.34 mm relative to the bregma was extracted using a mouse brain shape container. The striatum was dissected out using a scalpel blade. Tissue samples from the substantia nigra were taken with a tissue puncher of 2 mm diameter spanning across rostrocaudal levels − 2.70 to − 3.80 mm relative to the bregma. The samples were kept frozen until further analysis.

### Quantitative Immunohistochemistry

Immunohistochemistry and quantitative analyses were performed according to established protocols [17, 28] using primary antibodies against tyrosine hydroxylase [32] (rabbit anti-TH antiserum from Pel-Freez, Rogers, AR, 1:1000) and Cluster of Differentiation 68 (CD68) (rat anti-CD68 antiserum from AbD Serotec, Kidlington, Oxfordshire, UK; 1:1000). Quantitative analyses were performed by an experimentally blinded investigator using the following methods.

The number of TH-positive cell bodies in the substantia nigra compacta (SNc) was determined using unbiased stereology according to the optical fractionator method [33]. Analysis was performed using a Nikon 80i microscope with an *x*–*y* motorized stage controlled by the NewCAST software (Visiopharm, Hœrsholm, Denmark). The sampling fraction was chosen so as to count at least 100 neurons per side per animal following an established protocol [28], and the total number of TH-positive neurons in the SNc was then estimated using the optical fractionator formula, i.e., number of neurons = 1/ssf (slice sampling fraction) × 1/asf (area sampling fraction) × 1/tsf (thickness sampling fraction) × Σ (number of objects counted) [33].

The density of TH-immunoreactive fibers was measured in the highly denervated lateral part of the striatum using an image segmentation software (VIS, Visiopharm Integrator System; Visiopharm, Hørsholm, Denmark). To this end, the lateral striatum was outlined at low magnification (× 4 objective), using well-defined anatomical landmarks, in 4 rostrocaudal levels per animal (as in [28]). An automatic random sampling procedure was then applied under a ×100 objective to cover a fixed percentage of the structure of interest in all sections (10% of the lateral striatal cross-sectional area). Images were captured with a digital camera (Olympus DP72), obtaining 25 to 30 sample areas per mouse (area size, 5742 μm^2^). On each image, distinct TH-immunopositive fibers were separated from background objects using a Bayesian algorithm-based pixel classifier [34]. Results were expressed as the total number of TH-immunopositive pixels per sample area (averaged across areas). This analysis was not carried out in the sham-lesioned animals because of the fine dopaminergic fiber mesh present in this group (type I fibers, [35]), which precluded resolving distinct axonal structures. The high-magnification analysis of TH fibers on random sample areas was complemented by optical density (O.D.) measurements of TH immunostaining across the entire dorsolateral and ventrolateral striatum. Although this method is much less sensitive than the actual fiber analysis [17], it provides an indication of overall innervation density across the entire region of interest. The O.D. analysis was carried out in the same rostrocaudal levels using *NIH Image J Software*, as described in [28]. After subtracting background values (measured in the corpus callosum in each animal), the O.D. on the side ipsilateral to the lesion was expressed as a percentage of that on the intact side in the same animal (values from the intact side representing normal innervation densities [28]).

CD68-immunopositive cells exhibiting the morphology of active microglia [36, 37] were manually counted in the main body of the SNc (corresponding to rostrocaudal levels 3.40 to 3.64 mm posterior to the bregma, [38]) and ventrolateral striatum (0.26-0.02 mm anterior to the bregma, [38]). To this end, a mask delineating the region of interest was defined at × 4 magnification, and sample areas (36,921 μm^2^ in size) were randomly picked within this mask under a × 40 objective using an *x*–*y* motorized stage controlled by the NewCAST software (Visiopharm). The number of CD68-immunopositive cells on the side ipsilateral to the lesion was expressed as a percentage of that on the contralateral side.

### Western Blot Analysis

Tissue homogenates were prepared in cell lysis buffer (20 mmol/L Tris (pH 7.5), 150 mmol/L NaCl, 1 mmol/L EDTA, 1 mmol/L EGTA, 1% Triton X-100, 2.5 mmol/L sodium pyrophosphate, 1 mmol/L β-glycerolphosphate, 1 mmol/L Na_3_VO_4_, 1 μg/mL leupeptin, and 1 mmol/L phenylmethylsulfonyl fluoride). Twenty micrograms of protein was separated on a 10% SDS polyacrylamide gel. Proteins were transferred onto polyvinyldifluoride membranes, which were incubated in blocking buffer (20 mM Tris, 136 mM NaCl, pH 7.6, 0.1% Tween 20, 5% nonfat dry milk). Thereafter, membranes were incubated overnight at 4 °C using one of the following primary antibodies: rabbit polyclonal anti-GDNF (Santa Cruz Biotechnology, Inc., Santa Cruz, CA, 1:1000); monoclonal anti-BDNF (Santa Cruz Biotechnology, Inc., 1:1000); rabbit polyclonal against Thr202/Tyr204-phosphorylated ERK1/2 (p44/42-MAPK, Cell Signaling Technology Inc., Danvers, MA, 1:2000). Following appropriate washing steps, membranes were incubated with HRP-linked secondary antibodies (Sigma-Aldrich, Deisenhofen, Germany; 1:15000). Signals were visualized using a chemiluminescence kit (Merck Millipore, Watford, Hertfordshire, UK), and images were acquired using a CCD camera (LAS1000 system, Fuji Films, Tokyo, Japan). Optical density was measured on specific immunoreactive bands using *NIH Image J* software. Membranes were then stripped and reprobed with β-actin antibodies (Sigma-Aldrich, 1:50,000). The optical density of specific bands was then normalized to the β-actin band in the same lane.

### Pridopidine Brain Tissue Binding

Brain binding characteristics of pridopidine were evaluated *in vitro* using fresh mouse brain homogenates by rapid equilibrium dialysis using Rapid Equilibrium Dialysis (RED) Device inserts from ThermoScientific, according to the manufacturer’s protocol.

### Pharmacokinetics of Pridopidine in the Mouse

In a first pharmacokinetics (PK) study, pridopidine was given orally (p.o., which is the administration route used in humans). In this study, 4 doses of pridopidine (0.3, 3, 10, and 30 mg/kg, *N* = 24 per dose) were administered to male C57Bl6 mice for 7 days via oral gavage. Blood and brain samples were collected from 3 different mice per dose group per time point at the following times: prior to (*t* = − 10 min) and 15 min, 30 min, 1 h, 2 h, 4 h, 6 h, and 8 h post drug administration. A second study was carried out to compare the drug’s PK profile between oral and subcutaneous (s.c.) administration. Here, PK was examined following 7 daily administrations of 30 mg/kg pridopidine (HCl salt equivalents in water) either orally or subcutaneously (*N* = 24 mice per administration route). Blood and brain samples were collected from 3 animals per route per time point, at the following time points: predose, 15 min, 30 min, 1 h, 2 h, 4 h, 6 h, and 8 h post last dose.

Plasma samples were placed into K_3_-EDTA tubes (Becton-Dickinson, Mississauga, ON, Canada) and centrifuged for 5 min at 1500*g*_ave._. Brain tissue was collected and frozen without further process. Prior to analysis preweighed brain samples were diluted and homogenized with 0.01 M phosphate buffer containing 0.32 M sucrose, pH 7.4 in a ratio of 1 g of brain to 4 mL of homogenization solution.

### Quantification of Pridopidine Plasma and Brain Concentrations and Pharmacokinetic Analysis

Pridopidine and its internal standard, ACR354 (4-(3-methylsulfonyl)phenyl)-1-(propyld7)-piperidin-1-ium chloride), were extracted from EDTA plasma as well as from mouse brain homogenate (1:3 in phosphate-buffered saline, pH 7.4) by liquid–liquid extraction into acetonitrile. In brief, LC–MS/MS analyses were performed on a Shimadzu LC-20AD pump equipped with a Shimadzu SIL-20AC autosampler and Shimadzu CTO-20AC column oven. The MS/MS system was an MDS Sciex API-5000 mass spectrometer with an electrospray ionization probe (Toronto, Canada). Chromatographic separation of the analytes was achieved on Phenomenex, Synergi 2.5μ Hydro-RP column. The linearity was from 2 to 3000 ng/mL with a lower limit of quantification (LLOQ) of 2 ng/mL in plasma and 2 to 3000 ng/mL with LLOQ of 2 ng/mL in brain homogenate of 0.3 and 3 mg/kg-dose groups; 25 to 20,000 ng/mL in plasma and 75 to 60,000 ng/mL in brain homogenates, with LLOQ of 2 ng/mL and 75 ng/mL, respectively, for the higher doses. The deviation of pridopidine concentrations from quality control (QC) sample nominal concentration (accuracy values) was lower than 15% for all calibration curves. PK parameters were calculated using group mean concentration–time data, according to nominal time, that is, within ± 5% from the scheduled time point, by noncompartmental modeling for extravascular administration using WinNonlin 6.3. Dose-normalized exposure was used in order to estimate pridopidine exposure following 1 mg/kg dose (see Table 1).

**Table 1:** Plasma and brain exposure following pridopidine administration to C57Bl6 mice. Table shows concentrations of pridopidine in plasma and brain after administration of ascending pridopidine doses (0.3, 1, 3, 10, and 30 mg/kg). The maximal concentration (*C*_max_) is expressed as both nanograms per milliliter and micromolar. The AUC (area under curve, corresponding to the amount of pridopidine reaching the systemic circulation after a specific dose) is expressed as ng*hour/mL and in μM*hour. Each data point represents an average value obtained from 3 mice. Parameters for the 1 mg/kg dose are extrapolated based on average *C*_max_/dose and AUC_0-last_/dose values for 0.3, 3, 10, and 30 mg/kg groups. N.D.,  not determined.

Dose [mg/kg]	*C*_max_ [ng/mL]	*C*_max_ [μM]	AUC_0-last_ [ng*h*/mL]	AUC_0-last_ [μM*h]	*T*_max_ [h]
Plasma					
0.3	22	0.08	26	0.09	0.25
1^1)^	109	0.4	116	0.4	N.D.
3	277	1.0	406	1.4	0.5
10	1369	4.9	936	3.3	0.25
30	2577	9.2	3101	11.0	0.25
Brain					
0.3	110	0.39	120	0.43	0.25
1^1)^	331	1.2	392	1.4	N.D.
3	763	2.7	1051	3.7	0.25
10	3506	12.5	2971	10.6	0.5
30	6396	22.7	10,836	38.5	0.25

### Statistical Analysis

Behavioral data recorded over the chronic treatment period were compared using repeated-measures analysis of variance (ANOVA) and *post hoc* Bonferroni test. All remaining analyses were performed using either unpaired *t* test or 1-factor ANOVA and *post hoc* Tukey test. Striatal fiber densities were analyzed using nonparametric statistics (Kruskal–Wallis and *post hoc* Dunn’s test) because these data were not normally distributed. Unless otherwise stated, data are expressed as group mean ± standard error of the mean (SEM). The exact *p* and *F* values of the ANOVAs are reported in the figure legends, whereas *post hoc* pairwise comparisons are reported as being either significant or nonsignificant. In all comparisons, the level of statistical significance is set at *p* < 0.05.

## Results

### Pridopidine Induces Behavioral Recovery in 6-OHDA-Lesioned Mice

Wild-type mice sustaining intrastriatal 6-OHDA lesions or sham lesions were treated daily with pridopidine (0.3 or 1.0 mg/kg) or vehicle solution (saline) for 35 days starting on the same day of the lesion. In the final statistical analysis, sham-lesioned animals treated with pridopidine or saline were pooled in 1 group (“sham”) after ascertaining that they had yielded quite similar results.

**Fig. 1:**
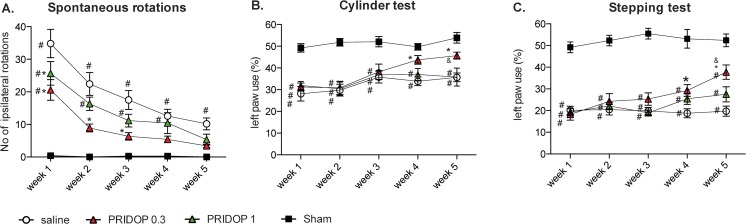
Behavioral improvement upon treatment with pridopidine. Spontaneous rotations (**A**) and forelimb use asymmetry (cylinder test, **B**, and stepping test, **C**) were assessed once a week in mice treated with either 0.3 or 1 mg/kg pridopidine (Pridop 0.3 or Pridop 1 mg/kg, respectively) or saline solution (*n* = 8-12 per group). Results are expressed as number of spontaneous ipsilateral rotations during a 10-min test session (**A**) or as percentage of supporting wall contacts (**B**) and adjusting steps (**C**) performed with the paw contralateral to the lesion (left paw). The lower dose of pridopidine (0.3 mg/kg) improved the animals’ performance in all the tests. The improvement in forelimb use (**B** and **C**) occurred gradually and became prominent after 5 weeks of treatment. Significant treatment effects were found in all these tests using repeated-measures ANOVA and *post hoc* Bonferroni test: (**A**) spontaneous rotations: ANOVA *p* < 0.0001 time, treatment, interaction; time *F*_(4,160)_ = 50.46, treatment *F*_(3,40)_ = 16.97, interaction *F*_(12,160)_ = 5.43; (**B**) cylinder test: ANOVA *p* < 0.0001 time and treatment,* p*> 0.05 interaction; time *F*_(4,160)_ = 8.74, treatment *F*_(3,40)_ = 20.96, interaction *F*_(12,160)_ = 1.52; (**C**) stepping test: ANOVA *p* = 0.0004 time, *p* < 0.0001 treatment, *p* = 0.006 interaction; time *F*_(4,160)_ = 5.46, treatment *F*_(3, 40)_ = 75.44, interaction *F*_(12,160)_ = 2.44; *p* < 0.05, asterisk, *versus* “saline”; number sign, *versus* “sham”; ampersand versus “Pridop 1”

A comparison of spontaneous turning behavior between groups and testing sessions revealed significant overall differences between treatments and time points (Fig. 1A). All groups injected with 6-OHDA exhibited a significant ipsilateral rotational bias, which was most pronounced on the first week and then gradually declined in all groups, as typically occurs with this test [17, 28]. There were, however, highly significant differences between groups in the extent of this decline (*p* < 0.0001 for the interaction between treatment and time, see Fig. 1A). Indeed, 6-OHDA-lesioned mice treated with saline maintained a significant difference from sham-lesioned animals from the first through the fifth and last week, whereas mice treated with pridopidine no longer differed from sham-lesioned controls by the end of the treatment period (Fig. 1 A, *p* > 0.05 for both drug doses *vs* sham on week 5). Furthermore, a difference was noticed between the 2 doses of pridopidine, as mice receiving the 0.3 mg/kg dose clearly diverged from saline-treated animals during most test sessions (*p* < 0.05 for pridopidine 0.3 mg/kg *vs* saline at weeks 1-3), whereas mice treated with 1.0 mg/kg pridopidine differed from the saline group only on the first test session (see week 1 in Fig. 1A, *p* < 0.05 for 1 mg/kg pridopidine *vs* saline).

In the cylinder test, the 6-OHDA lesion produced a similar deficit in contralateral forelimb use in all treatment groups (see data from week 1 in Fig. 1B, ~ 40% difference and *p* < 0.05 *vs* sham in both saline- and pridopidine-treated mice). This significant difference from sham-lesion controls was maintained in all groups through the third week (Fig. 1B). Thereafter, mice treated with 0.3 mg/kg pridopidine showed a visible improvement, reaching levels of performance comparable with sham-control values on weeks 4 and 5 (Fig. 1B, week 4, *p* < 0.05 *vs* saline and *p* > 0.05 *vs* sham; week 5, *p* < 0.05 *vs* both saline and 1.0 mg/kg dose and *p* > 0.05 *vs* sham). In contrast, treatment with 1.0 mg/kg pridopidine did not restore contralateral forelimb use in the cylinder test (see week 5 in Fig. 1B, ~ 33% difference and *p* < 0.05 *vs* sham in both pridopidine-1 and saline group).

Significant overall differences between groups and testing sessions were also found in the stepping test (Fig. 1C). The lesion-induced stepping deficit was initially severe in all groups (see data from week 1 in Fig. 1C, ~ 60% difference and *p* < 0.05 *vs* sham in both saline- and pridopidine-treated mice). All 6-OHDA-lesioned mice continued to show a significant deficit during the first 2 weeks regardless of treatment allocation. Thereafter, animals treated with 0.3 mg/kg pridopidine showed a gradual improvement, diverging significantly from the saline-treated group on weeks 4 and 5. Although not reaching sham-control values, the performance of mice treated with 0.3 mg/kg pridopidine had improved by ~ 94% above the vehicle group (saline) by the end of the treatment period (see week 5 in Fig. 1C, *p* < 0.05 for pridopidine-0.3 *vs* both saline and pridopidine-1). Animals receiving 1.0 mg/kg pridopidine showed a trend towards an improved stepping performance during the last 2 weeks, but the difference from saline-treated mice did not reach significance (Fig. 1C, cf. pridopidine-1 and saline group in week 5, ~ 49% and ~ 63% difference *vs* sham, respectively, *p* < 0.05).

### Pridopidine Has Neuroprotective and Neurorestorative Effects on Nigrostriatal Dopamine Neurons

**Fig. 2:**
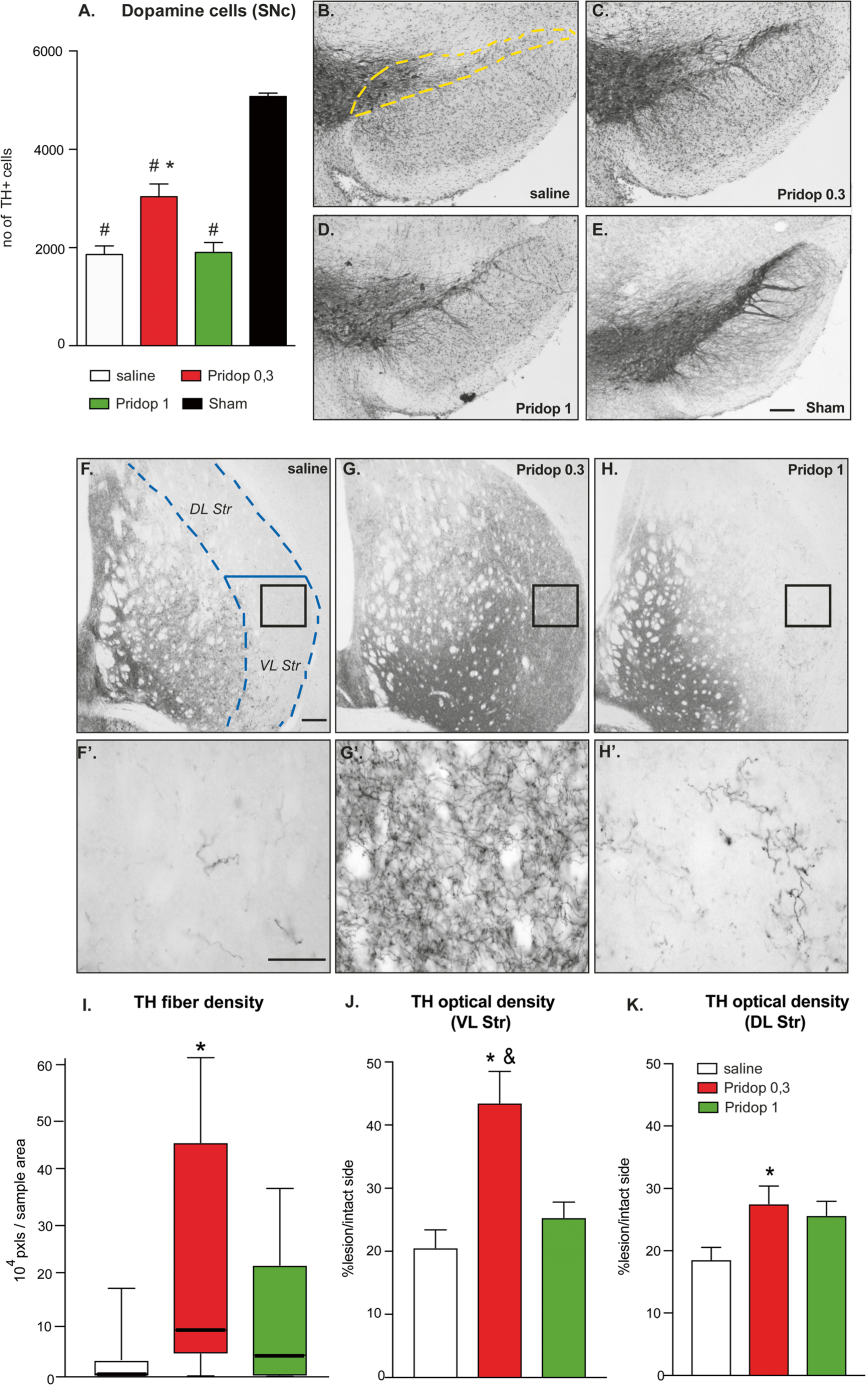
Chronic treatment with pridopidine induces neurohistological restoration. (**A**) Stereological counts of TH-positive cells were performed in the substantia nigra pars compacta (SNc) (the dashed yellow line outlines the area included in the analysis). Data show the total number of cells from the SNc ipsilateral to the lesion (counted on 7 sections per animal throughout the SNc). One-way ANOVA *p* < 0.0001; *F*_(3,39)_ = 44.29; *post hoc* Tukey test, *p* < 0.05 asterisk *versus* saline; number sign, *versus* sham. (**B**-**E**) Low-magnification photomicrographs of nigral sections immunostained for TH, representing the different groups. Scale bar 100 μm. (**F**-**H**) Low-magnification photomicrographs of striatal sections; the dashed blue line represents the area included in the analysis of TH fiber density. (**F′**-**H′**) High-magnification insets from the same sections illustrate differences in fiber density and morphology between the different groups (**F′**, saline-treated case; **G′**, mouse treated with 0.3 mg/kg pridopidine, **H**′, mouse treated with 1 mg/kg pridopidine). Scale bar, 200 μm. (**I**) Image segmentation analysis of TH-positive fibers as performed at high magnification on random sample areas. Values give fiber pixels/sample area, whiskers encompass the entire range of values in each group (the median is shown as a horizontal line). Kruskal–Wallis and *post hoc* Dunn’s test, *p* = 0.0091; asterisk, *p* < 0.05 *versus* saline. (**J**-**K**) Optical density analysis of TH immunostaining over the entire cross-sectional area of the ventrolateral (**J**) or dorsolateral (**K**) striatum; O.D. values from the side ipsilateral to the lesion are expressed as a percentage of those from the contralateral intact side in each animal. (**J**) Ventrolateral (VL) striatum, ANOVA *p* = 0.0002, *F*_(2,33)_=11.35; (**K**) Dorsolateral (DL) striatum, ANOVA *p* = 0.039, *F*_(2,33)_ = 3.56. *Post hoc* Tukey test, *p* < 0.05 asterisk, *versus* saline; ampersand, *vs* Pridop 1

Unbiased stereology was used to count dopaminergic cell bodies in the SNc. On the side ipsilateral to the lesion, mice treated with saline displayed ~ 65% reduction in nigral TH-positive neurons (i.e., ~ 1960 cells in saline-treated mice *vs* ~ 5076 cells in sham-lesion controls, *p* < 0.05, Fig. 2A). Mice treated with 0.3 mg/kg pridopidine showed a significantly larger number of TH-positive neurons (~ 3034 cells), corresponding to an increase by ~ 55% relative to saline treatment (Fig. 2A, *p* < 0.05). The higher dose of pridopidine (1.0 mg/kg) did not, however, confer significant protection of dopaminergic cell bodies in SNc (~ 1901 cells counted; *p* > 0.05 *vs* saline), although it appeared to produce a larger density of TH-immunopositive neurites in the SN pars reticulata (cf. Fig 2B and D).

To examine the effects of pridopidine treatment on striatal dopamine (DA) innervation, we first measured TH-immunopositive axon fibers at high magnification by randomly sampling an estimated 10% of the cross-sectional area over the lateral caudate–putamen (encased by hatched lines in Fig. 2F). This analysis could not be applied to sham-lesioned mice due to the impossibility to resolve distinct fibers in an intact striatum [17, 35, 39]. Chronic treatment with 0.3 mg/kg pridopidine markedly enhanced the number of TH fiber profiles (cf. Fig. 2F and G), yielding an increase by about 15-fold in median values relative to those of saline-injected animals (Fig. 2I, *p* < 0.05). All animals treated with pridopidine 0.3 mg/kg showed an abundance of thick and highly branched axons harboring enlarged varicosities (Fig. 2G′), indicative of a sprouting response [35, 39]. Although clearly less abundant, similar morphological features were also encountered in mice treated with 1.0 mg/kg pridopidine (Fig. 2H-H′), but the effect of this larger dose did not achieve statistical significance (Fig. 2I, *p* > 0.05 for pridopidine-1 *vs* saline).

A gross estimate of DA innervation levels was obtained by measuring the O.D. of TH immunostaining in the ventrolateral and dorsolateral striatum through the entire extent of these regions (Fig. 2J, K). In the ventrolateral striatum, treatment with 0.3 mg/kg pridopidine yielded an over twofold increase in TH O.D. above saline control levels (*p* < 0.05) (Fig. 2J). Mice treated with 1 mg/kg pridopidine showed a trend towards an increase that did not reach statistical significance (Fig. 2J). Animals treated with 0.3 pridopidine showed a significant, albeit smaller increase in TH O.D. also in the dorsolateral striatum, while animals treated with 1 mg/kg pridopidine showed, again, only a trend (Fig. 2K).

Taken together, these results indicate that treatment with 0.3 mg/kg pridopidine had provided partial protection of nigral DA cell bodies, accompanied by a marked dopaminergic reinnervation of the highly depleted lateral striatum. The latter effect was particularly prominent in the ventrolateral quadrant, a region controlling forelimb use in rodents [40, 41]. Despite several positive trends, the larger dose of pridopidine (1 mg/kg) did not achieve significant effects on any parameter.

**Fig. 3:**
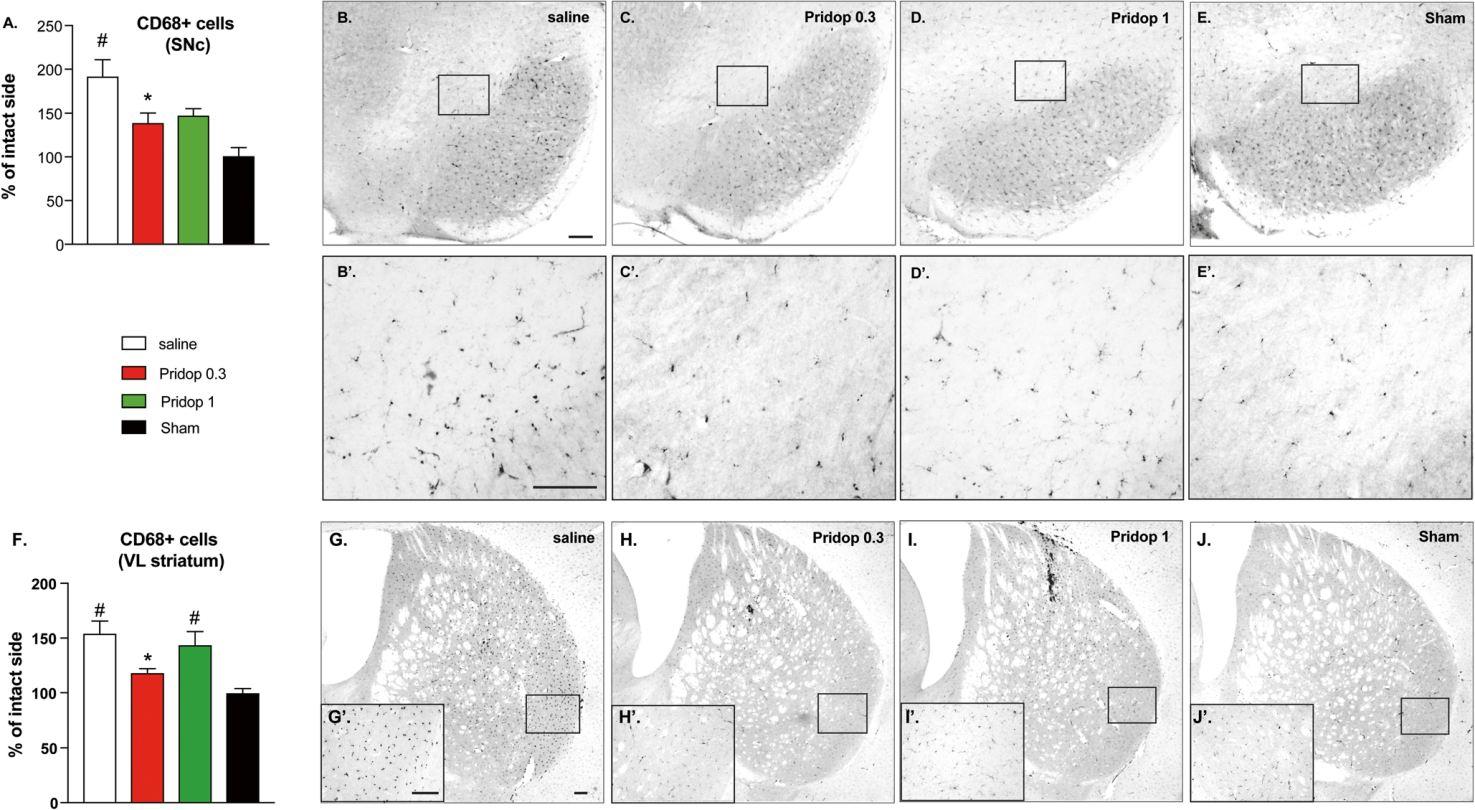
Pridopidine treatment reduces microglia activation in the nigrostriatal pathway. Counts of CD68-positive microglial cells in the SNc (**A**) and ventrolateral striatum (**F**) and corresponding photomicrographs (**B**-**E**' and **G**-**J**', respectively). Mice receiving 0.3 mg/kg pridopidine showed a significantly lower number of CD68-positive cells compared to saline-treated controls at both nigral and striatal levels. Low-magnification photomicrographs (**B**-**J**) were taken under a ×4 objective, whereas insets (**B′**-**J′**) were photographed under a ×20 objective. Scale bars, 100 μm. (**A**) One-way ANOVA *p* = 0.0002, *F*_(3,28)_ = 9.005; (**F**) one-way ANOVA *p* = 0.0005, *F*_(3,28)_ = 8.049. *Post hoc* Tukey test, *p* < 0.05 asterisk, *versus* saline; number sign, versus sham

We next asked whether the neurorestorative effects of pridopidine treatment may have coincided with a reduced microglial activation in the involved regions. To this end, we counted the number of CD68-immunopositive microglial cells in the SNc and the ventrolateral striatum. This number was significantly reduced in both regions in mice treated with 0.3 mg/kg pridodipine (Fig. 3A, F; *p* < 0.05 for pridopidine-0.3 *vs* saline, *p* > 0.05 *vs* sham). Mice treated with the larger dose of pridopidine showed a trend towards an effect in the SNc (Fig. 3A; *p* > 0.05 *vs* sham), but not in the ventrolateral striatum (Fig. 3F; *p* < 0.05 *vs* sham, *p* > 0.05 *vs* saline).

### Treatment with Pridopidine Is Not Effective in Sigma-1 KO Mice

**Fig. 4:**
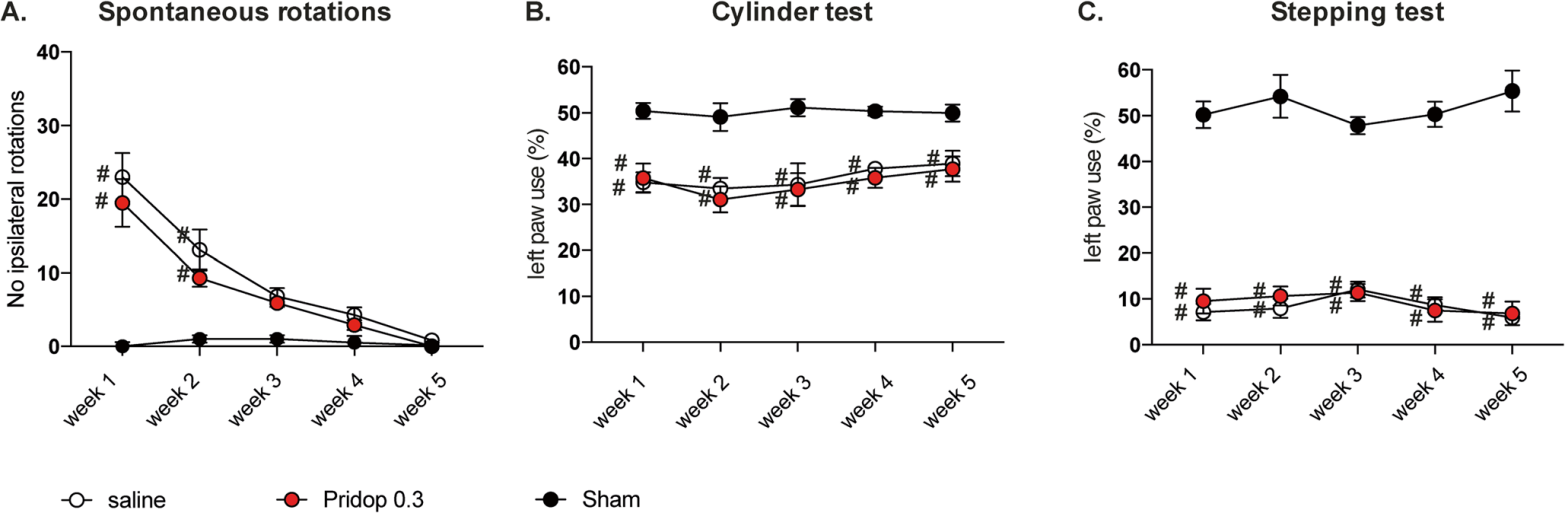
Pridopidine does not improve motor deficits in 6-OHDA-lesioned mice that lack S1R. Spontaneous rotations (**A**) and forelimb use asymmetry (cylinder test, **B**, and stepping test, **C**) were assessed once a week in S1R KO mice sustaining 6-OHDA lesions or sham lesions, followed by treatment with either 0.3 mg/kg pridopidine (Pridop 0.3) or saline solution (*n* = 8-10 per group). Results are expressed as number of spontaneous ipsilateral rotations during a 10-min test session (**A**) or as percentage of supporting wall contacts (**B**) and adjusting steps (**C**) performed with the paw contralateral to the lesion (left paw). Repeated-measures ANOVA and *post hoc* Bonferroni test: (**A**) spontaneous rotations: ANOVA *p* < 0.0001 time, treatment, interaction, time *F*_(4,104)_ = 45.36, treatment *F*_(2,26)_ = 15.54, interaction *F*_(8,104)_ = 11.15; (**B**) cylinder test: ANOVA *p* > 0.05 time and interaction, *p* < 0.0001 treatment, time *F*_(4,104)_ = 1.23, treatment *F*_(2,26)_ = 1.76, interaction *F*_(8,104)_ = 0.32; (**C**) stepping test: ANOVA *p* > 0.05 time and interaction, *p* < 0.0001 treatment, time *F*_(4,104)_ = 0.46, treatment *F*_(2,26)_ = 346.4, interaction *F*_(8,104)_ = 1.35; *p* < 0.05, number sign *versus* “sham”

**Fig. 5:**
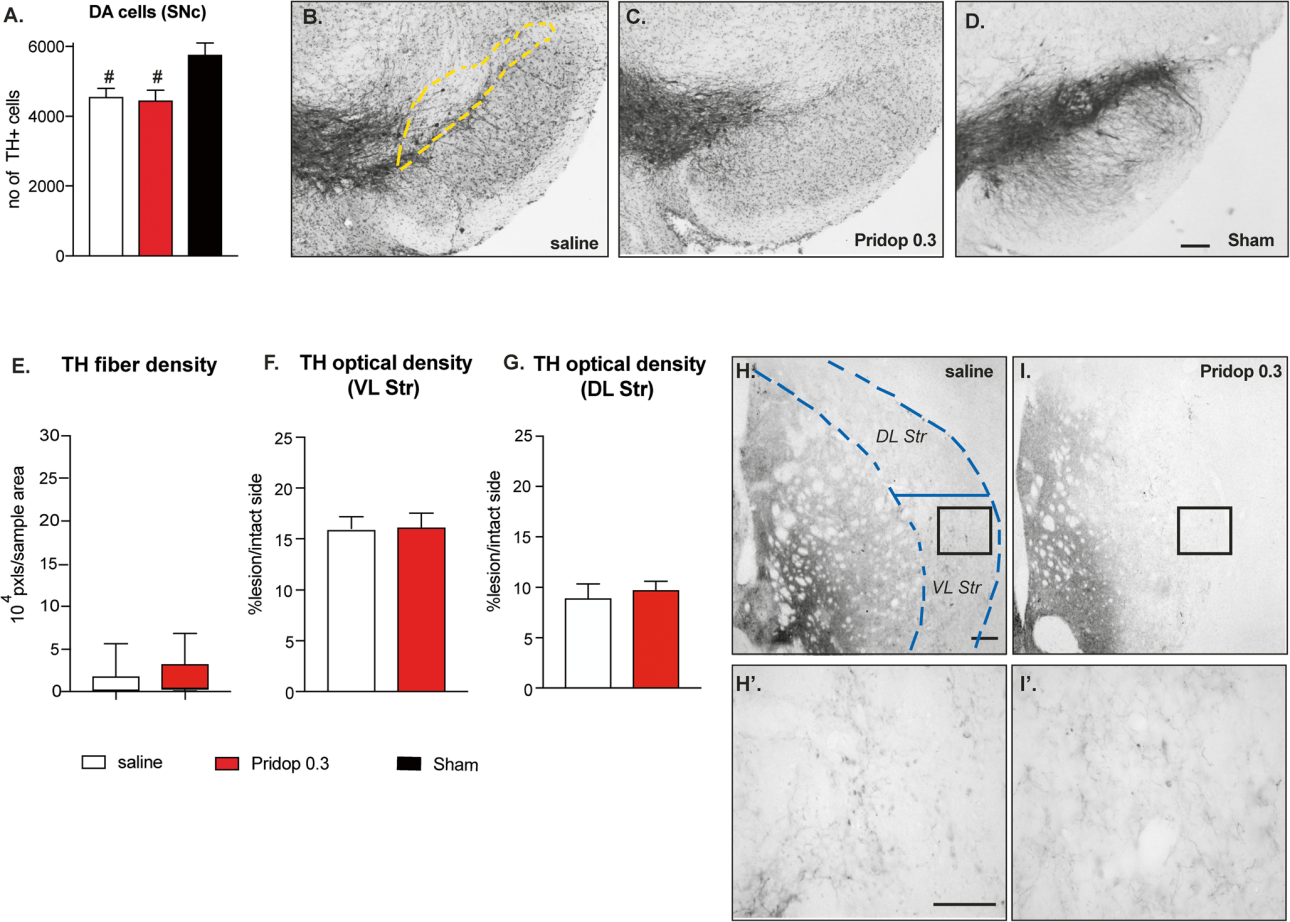
Pridopidine is devoid of neurorestorative effects in 6-OHDA-lesioned mice that lack S1Rs. (**A**) Stereological counts of TH-positive cells in the SNc in S1R KO mice and (**B**-**D**) low-magnification photomicrographs of nigral sections immunostained for TH (the dashed yellow line outlines the area included in the analysis). Scale bar, 100 μm. Data in A show the total number of cells from the SNc ipsilateral to the lesion (counted on 7 sections per animal throughout the SNc). One-way ANOVA *p* < 0.0001, *F*_(2,26)_ = 41.73; *post hoc* Bonferroni test; *p* < 0.05 number sign *versus* sham group. (**E**) Image segmentation analysis of TH-positive fibers as performed at high magnification on random sample areas through the lateral striatum (encased by dashed blue lines). Values are expressed as fiber pixels/sample area, whiskers encompass the entire range of values in each group, the median is shown as a horizontal line. Mann–Whitney test, *p* = 0.084. (**F**-**G**) Optical density (O.D.) of TH immunostaining over the entire cross-sectional area of ventrolateral (**F**) and dorsolateral (**G**) striatum. O.D. values from the side ipsilateral to the lesion are expressed as a percentage of those from the contralateral intact side in each animal. (**F**) Ventrolateral (VL) striatum, unpaired *t* test, *p* = 0.516; (**G**) Dorsolateral (DL) striatum, *p* = 0.219. (**H**-**I′**) Representative photomicrographs of TH-immunostained striatal sections from a saline-treated (**H**, **H′**) and a pridopidine-0.3 mg/kg treated S1R KO mouse (**I**, **I′**). Scale bar, 200 μm

We next evaluated the effective dose of pridopidine (0.3 mg/kg) in S1R KO mice sustaining 6-OHDA lesions or sham lesions. S1R KO-lesioned mice treated with either 0.3 mg/kg pridopidine or saline showed similar levels of spontaneous rotations (Fig. 4A) and forelimb use asymmetry in cylinder test (Fig. 4B) and stepping test (Fig. 4C) throughout the 5 weeks of treatment (*p* > 0.05 for pridopidine *vs* saline in all tests and sessions). Moreover, treatment with pridopidine did not have any effect on either nigral DA neurons (Fig. 5A) or striatal TH fiber density (Fig. 5E-G) (*p* > 0.05 for pridopidine *vs* saline on all parameters). These data clearly indicate that, in the absence of S1R, pridopidine cannot exert any restorative action on the damaged nigrostriatal DA pathway.

An interesting collateral finding of this experiment is that S1R KO mice had responded to the 6-OHDA lesion with a distinctive pattern of nigrostriatal damage. Indeed, compared to wild-type animals, 6-OHDA-lesioned S1R KO mice featured a milder loss of DA cell bodies in the SNc (~ 22% instead of ~ 61% loss, cf. saline groups in Figs. 2A and 5A), but a lower density of TH fibers in the lateral striatum (median value of 899 and 5842 fiber pixels/sample area in KO and wild-type mice, respectively; cf. saline groups in Figs. 2I and 5E). A more severe degeneration of striatal dopaminergic fibers compared to nigral cell bodies in S1R KO mice is likely to account for apparent differences in lesion-induced motor impairments relative to the wild-type genotype, in particular, the less severe rotational bias concurring with a more severe deficit in forelimb stepping (cf. Figs. 1 and 4; see “Discussion” for comments on the significance of these motor features).

### Pridopidine Upregulates Neurotrophic Factors and Activates ERK1/2 in the Striatum

**Fig. 6:**
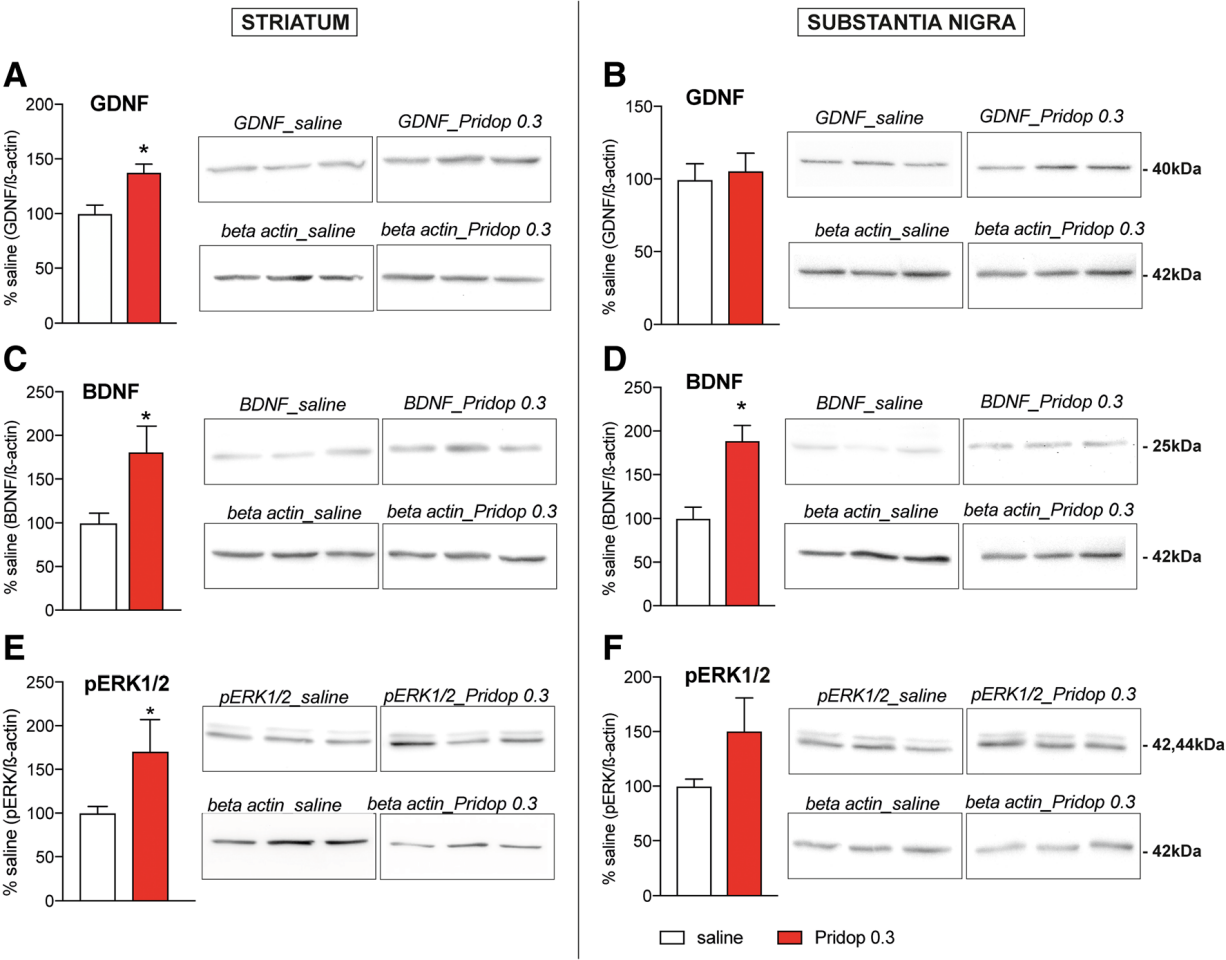
Treatment with 0.3 mg/kg pridopidine upregulates trophic factors and phosphorylated ERK1/2. Western blot analysis of GDNF, BDNF, and phosphorylated ERK1/2 (pERK1/2) using striatal samples (**A**, **C**, **E**) and nigral samples (**B**, **D**, **F**) from 6-OHDA-lesioned wild-type mice treated with 0.3 mg/kg pridopidine (*n* = 9) or saline solution (*n* = 11) for 5 weeks. Results are normalized for β-actin levels, and data from pridopidine-treated animals are shown as a percentage of the values from saline-treated controls. Unpaired *t* test, striatum: (**A**) GDNF *p* = 0.003; (**B**) BDNF *p* = 0.013, (**C**) pERK1/2 *p* = 0.049; substantia nigra: (**D**) GDNF *p* = 0.72; (**E**) BDNF *p* = 0.0008; (**F**) pERK1/2 *p* = 0.106

The gradual improvement in forelimb use and the concomitant increase in striatal DA fibers density induced by pridopidine 0.3 mg/kg are indicative of a neurorestorative action mediated by trophic factors, whose brain levels can increase upon pharmacological stimulation of S1R [10, 17, 42]. Western blot analysis of GDNF, BDNF, and phosphorylated ERK1/2 (a major intracellular effector of neurotrophin signaling [43]) was therefore carried out in additional groups of wild-type mice sustaining intrastriatal 6-OHDA lesions followed by a 35-day treatment with 0.3 mg/kg pridopidine or saline. Pridopidine promoted a significant striatal upregulation of GDNF and BDNF levels (+ 37.6% and + 80.8% *vs* saline, respectively, *p* < 0.05 for both markers, Fig. 6A, C). In the SN, only BDNF levels showed a significant increase (+ 88.5% *vs* saline, Fig. 6D, *p* < 0.05). Treatment with pridopidine 0.3 mg/kg also produced an increase in striatal and nigral levels of phosphorylated ERK1/2 (Fig. 6E, F), which, however, reached significance only in the striatum (+ 71% *vs* saline, *p* < 0.05, Fig. 6E).

### Pridopidine Pharmacokinetics in Mice

Pridopidine presented similar plasma and brain PK following oral and subcutaneous single-dose administrations (Supplemental Table S1), with a relative oral/subcutaneous exposure ratio of 117% for *C*_max_ and 90% for AUC_0-*t*_ in plasma, and 88% for *C*_max_ and 96% for AUC_0-*t*_ in brain tissue. Thus, pridopidine exposure data following oral dosing fairly represent the exposure achieved by subcutaneous administration. A dose–response PK study after oral administration indicated that the drug’s plasma and brain exposure was dose-proportional (Table 1). Brain-to-plasma ratio measurements showed that pridopidine concentrations were four- to fivefold larger in the brain than in plasma for the 0.3 mg/kg dose and two- to threefold larger for the dose range 1 to 30 mg/kg (cf. *C*_max_ and AUC values in the brain *vs* plasma, Table 1). Administration of 0.3 and 1 mg/kg pridopidine yielded brain *C*_max_ values of 110 and 331 ng/mL (corresponding to 390 nM and 1200 nM), respectively, occurring at a median *T*_max_ of 0.25 h and with corresponding AUC_0-last_ values of 120 and 392 ng*h/mL (corresponding to 0.43 and 1.4 h*μM) (Supplemental Table S1). Pridopidine was rapidly cleared, with brain total levels close to 3 ng/mL (corresponding to 11 nM, out of which only ~ 2 nM are free) already after 3 h.

The free fraction of pridopidine was determined using a commercially available *in vitro* protein binding assay. At the doses of 0.3 and 1 mg/kg, the unbound (free) fraction of pridopidine in brain tissue was estimated to be ~ 50% (Supplemental Table S2). Therefore, brain free pridopidine concentrations at *C*_max_ were estimated to correspond to 55 and 165.5 ng/mL (or 195 nM and 600 nM) for the doses of 0.3 and 1 mg/kg, respectively. Based on the reported binding affinity of pridopidine to the rat S1R (Ki 69.7 nM, [25]), it thus seems plausible that, at *C*_max_, both pridopidine doses would yield 100% occupancy of the S1R.

## Discussion

Pridopidine was initially developed to treat motor symptoms caused by altered dopaminergic transmission. The motor effects of pridopidine were originally attributed to its low-affinity, antagonistic activity at the D2R [19, 26]. However, such an activity cannot fully account for the action of pridopidine, as indicated by behavioral studies in mice lacking the D2R [44] and by *in vivo* D2R binding studies in rats [27]. Pridopidine has high binding affinity to S1R, with a reported Ki of 69.7 nM for rat S1R and 81.7 nM for the human S1R [25]. Recent studies indicate that it can exert neuroprotective effects via this target. In particular, pridopidine has synaptoprotective effects in a cellular model of HD, rescuing dendritic spine impairment and normalizing aberrant calcium signaling via the S1R [45]. Pridopidine has also been found to enhance the secretion of BDNF in a neuroblastoma cell line in a S1R-dependent manner [46].

The present study has addressed the hypothesis that pridopidine can exert neuroprotective and neurorestorative effects via the S1R in an animal model of PD. To this end, we have utilized the same PD model and experimental design as in our previous study of the selective S1R agonist, PRE-084 [17]. In this animal model, microinjections of 6-OHDA are applied to the lateral part of the caudate–putamen in order to induce a retrograde degeneration of dopaminergic neurons projecting to this region. The procedure yields a very severe depletion of dopaminergic fibers in the motor part of the striatum, with substantial fiber sparing in the most medial, associative-limbic regions, thus resembling the denervation pattern characteristic of early-stage PD [47]. Another interesting feature of this animal model is the occurrence of inflammatory reactions at both striatal and nigral levels, which contribute to maintaining and amplifying the neurodegenerative process [36, 48–50], similarly to the situation in PD [14]. Finally, this model features multiple motor deficits that are caused by DA depletion and can thus report the extent of dopaminergic restoration achieved by different treatments [13, 17, 51–53]. For all the above reasons, mice with intrastriatal 6-OHDA lesions were the model of choice in the present study, even though we acknowledge the limitations of this animal model in terms of lacking protein aggregation pathology.

Our results show that pridopidine exerts a profile of effects quite similar to that produced by PRE-084 in a previous study [17]. Like PRE-084, pridopidine was effective at the dose of 0.3 mg/kg, which countered the lesion-induced rotational asymmetry and induced a gradual improvement in forelimb use in both cylinder and stepping tests. These behavioral effects were paralleled by a significant protection of DA cell bodies in the SNc and a marked restoration of TH fiber density in the ventrolateral striatum, the region controlling forelimb use in rodents [40, 41]. The higher dose of pridopidine (1 mg/kg) had beneficial effects on the lesion-induced rotational bias, although it was overall ineffective on most behavioral and neurohistological endpoints. This finding too resembles the results obtained with the S1R agonist PRE-084, which was found to ameliorate spontaneous turning behavior without improving forelimb use when given at the dose of 1 mg/kg [17]. A definite proof that the neurorestorative effects of 0.3 mg/kg pridopidine depended on S1R was obtained by evaluating this dose in S1R KO mice lesioned with 6-OHDA. In this case, no difference was found between pridopidine and vehicle (saline) in any behavioral or neurohistological parameter. Interestingly, the study of S1R KO mice revealed some previously unappreciated effects of Sigmar1 genetic ablation on the response to 6-OHDA. Indeed, S1R KO mice showed a less severe loss of DA cell bodies in the SNc but a more severe depletion of striatal DA fibers than did wild-type animals. S1R KO mice have been found to express lower levels of the DA transporter (DAT) [54], which is implicated in the uptake of 6-OHDA [55]. While lower DAT levels might have mitigated the neurotoxic insult to DA neurons, the particularly severe striatal denervation observed in KO mice indicates that S1R deficiency had increased the vulnerability to axonal degeneration in the nigrostriatal pathway. This interpretation appears consistent with the known functions of S1R in regulating the growth and repair of neuritic processes, as reported in several types of cells and models [1, 8].

The superior efficacy of a lower pridopidine dose (0.3 mg/kg) compared to a larger one (1.0 mg/kg) is in keeping with results obtained using potent S1R agonists both in 6-OHDA-lesioned mice [17] and other mouse models of neurodegenerative disease [5, 10, 56]. This observation appears consistent with the bell-shaped dose–response curve reported for high-affinity S1R agonists [6, 9, 57]. To explain this bell-shaped profile, it has been proposed that larger concentrations of S1R agonists may have off-target effects (e.g., on sigma-2 sites) opposing their beneficial properties [20]. In our study, however, there was no indication that pridopidine 1 mg/kg might have opposed a S1R-dependent neurorestoration. Indeed, this drug dose improved the animals’ rotational bias and tended to improve most neurohistological endpoints. The low efficacy of this higher dose might instead reflect a suboptimal modulation of S1R dynamics. Indeed, S1R traffics across subcellular compartments and interacts with various proteins in a ligand-dependent manner [58, 59], and it is therefore likely that the receptor occupancy by exogenous ligands should not exceed a critical window in order to provide therapeutic benefit. Further studies aimed at substantiating this hypothesis are clearly warranted.

Although it was already known that S1R agonists have a bell-shaped dose–response curve, our PK study contributes novel valuable information as to the relationship between effective doses and brain exposure. Thus, our data show that the brain free fraction of 0.3 mg/kg pridopidine (able to bind to the target) reached a maximal concentration (*C*_max_) of 55 ng/mL (195 nM), which is ~ 2.7-fold the drug’s Ki for rat S1R (~ 69.7 nM). Pridopidine reached *C*_max_ already after ~ 25 min and was quickly cleared, with negligible levels already 3 h post drug administration. These data therefore suggest that drug doses occupying brain S1Rs for 1-2 h per day are sufficient to achieve neuroprotective and neurorestorative effects, at least in this animal model of nigrostriatal degeneration. However, this suggestion needs to be verified directly using *in vivo* S1R occupancy assays.

Finally, the present study offers some clues about the mechanisms underlying the functional neurorestoration induced by pridopidine in this parkinsonian model. The effect profile of the lower drug dose (0.3 mg/kg) includes both protection of nigral DA cell bodies and restoration of dopaminergic fiber density in the lateral striatum. In this region, the TH-positive fiber network exhibited morphological features typical of regenerating axons [35, 39]. Along with these morphological observations, the gradual restoration of forelimb use (which became significant only after 4 weeks of pridopidine treatment) points to a stimulation of DA axon terminal sprouting as a pivotal mechanism of functional recovery. This interpretation is further supported by the significant upregulation of GDNF, BDNF, and phosphorylated ERK1/2 produced by 0.3 mg/kg pridopidine in the striatum. These factors strongly stimulate neuritic outgrowth from nigrostriatal DA neurons both *in vitro* and *in vivo* (reviewed in [13]). The upregulation of GDNF, BDNF, and phosphorylated ERK1/2 by pridopidine treatment is in keeping with the effects produced by 0.3 mg/kg PRE-084 in our previous study [17], and it is also consistent with other studies showing glial upregulation of GDNF [10] or increased posttranslational processing and release of BDNF [42] by S1R agonists. Based on previous studies in 6-OHDA-lesioned rodents [17, 40, 60], the extent of DA reinnervation achieved by pridopidine 0.3 mg/kg in the ventrolateral striatum can be deemed sufficient to improve forelimb use even if a normal fiber density was not restored.

The neuroprotective effect of pridopidine at the nigral level occurred already during the first week of treatment, as indicated by a prompt improvement of the lesion-induced rotational bias. Indeed, the rotational behavior of unilateral 6-OHDA-lesion models appears to depend more on nigral than striatal mechanisms [31, 61, 62]. Supporting this interpretation, we have previously found that treatment with PRE-084 0.3 mg/kg protected nigral DA neurons to a similar extent upon short- and long-duration treatment (7 and 35 days, respectively), although it restored impairments in forelimb use and striatal DA fiber densities only after the longer treatment [17]. Neuroprotection at the nigral level was attributed to an early reduction in microglia activation, as detected in the SNc already after 7 days of treatment with PRE-084 0.3 mg/kg [17]. Although shorter treatment periods with pridopidine were not investigated, our results show that the extent of microglial activation in the SNc was significantly reduced after 5 weeks of treatment with the 0.3 mg/kg dose. In addition to this anti-inflammatory effect, the treatment-induced upregulation of nigral BDNF levels was certainly beneficial to the survival of DA neurons (reviewed in [13]).

In conclusion, the present study provides the first demonstration that pridopidine behaves like a S1R agonist *in vivo*, producing functionally relevant neuroprotective and neurorestorative effects that are typical for high-affinity S1R agonists in this mouse model of PD. Pridopidine is a clinically tested compound with an excellent safety and tolerability profile [22, 63, 64]. Our results provide a rationale for further investigations of pridopidine as a potential disease-modifying treatment in parkinsonian disorders.

## Electronic Supplementary Material

ESM 1 (PDF 1225 kb)

ESM 2 (PDF 1225 kb)

ESM 3 (PDF 1225 kb)

ESM 4 (PDF 1225 kb)

ESM 5 (PDF 1197 kb)

ESM 6 (PDF 1225 kb)

ESM 7 (PDF 1225 kb)

ESM 8 (DOCX 21 kb)

## References

[1] Hayashi T, Su TP (2007). Sigma-1 receptor chaperones at the ER-mitochondrion interface regulate Ca(2+) signaling and cell survival. Cell..

[2] Ajmo CT, Vernon DO, Collier L, Pennypacker KR, Cuevas J (2006). Sigma receptor activation reduces infarct size at 24 hours after permanent middle cerebral artery occlusion in rats. Current Neurovascular Research.

[3] Harukuni I, Bhardwaj A, Shaivitz AB, DeVries AC, London ED, Hurn PD (2000). Sigma(1)-receptor ligand 4-phenyl-1-(4-phenylbutyl)-piperidine affords neuroprotection from focal ischemia with prolonged reperfusion. Stroke..

[4] Lahmy V, Meunier J, Malmstrom S, Naert G, Givalois L, Kim SH (2013). Blockade of Tau hyperphosphorylation and Abeta(1)(−)(4)(2) generation by the aminotetrahydrofuran derivative ANAVEX2-73, a mixed muscarinic and sigma(1) receptor agonist, in a nontransgenic mouse model of Alzheimer’s disease. Neuropsychopharmacology : official publication of the American College of Neuropsychopharmacology..

[5] Mancuso R, Olivan S, Rando A, Casas C, Osta R, Navarro X (2012). Sigma-1R agonist improves motor function and motoneuron survival in ALS mice. Neurotherapeutics : the journal of the American Society for Experimental NeuroTherapeutics..

[6] Prause J, Goswami A, Katona I, Roos A, Schnizler M, Bushuven E (2013). Altered localization, abnormal modification and loss of function of Sigma receptor-1 in amyotrophic lateral sclerosis. Human molecular genetics..

[7] Ruscher K, Inacio AR, Valind K, Rowshan Ravan A, Kuric E, Wieloch T (2012). Effects of the sigma-1 receptor agonist 1-(3,4-dimethoxyphenethyl)-4-(3-phenylpropyl)-piperazine dihydro-chloride on inflammation after stroke. PloS one..

[8] Ruscher K, Shamloo M, Rickhag M, Ladunga I, Soriano L, Gisselsson L (2011). The sigma-1 receptor enhances brain plasticity and functional recovery after experimental stroke. Brain : Journal of Neurology..

[9] Villard V, Espallergues J, Keller E, Vamvakides A, Maurice T (2011). Anti-amnesic and neuroprotective potentials of the mixed muscarinic receptor/sigma 1 (sigma1) ligand ANAVEX2-73, a novel aminotetrahydrofuran derivative. Journal of Psychopharmacology..

[10] Penas C, Pascual-Font A, Mancuso R, Fores J, Casas C, Navarro X (2011). Sigma receptor agonist 2-(4-morpholinethyl)1 phenylcyclohexanecarboxylate (Pre084) increases GDNF and BiP expression and promotes neuroprotection after root avulsion injury. Journal of Neurotrauma..

[11] Hong J, Wang L, Zhang T, Zhang B, Chen L (2017). Sigma-1 receptor knockout increases alpha-synuclein aggregation and phosphorylation with loss of dopaminergic neurons in substantia nigra. Neurobiology of Aging..

[12] Lang AE, Espay AJ (2018). Disease modification in Parkinson’s disease: current approaches, challenges, and future considerations. Movement disorders : Official Journal of the Movement Disorder Society..

[13] Francardo V, Schmitz Y, Sulzer D, Cenci MA (2017). Neuroprotection and neurorestoration as experimental therapeutics for Parkinson’s disease. Experimental Neurology..

[14] Tansey MG, Goldberg MS (2010). Neuroinflammation in Parkinson’s disease: its role in neuronal death and implications for therapeutic intervention. Neurobiology of Disease..

[15] Allen Reish HE, Standaert DG (2015). Role of alpha-synuclein in inducing innate and adaptive immunity in Parkinson disease. Journal of Parkinson's Disease..

[16] Surmeier DJ, Schumacker PT, Guzman JD, Ilijic E, Yang B, Zampese E (2017). Calcium and Parkinson’s disease. Biochemical and Biophysical Research Communications..

[17] Francardo V, Bez F, Wieloch T, Nissbrandt H, Ruscher K, Cenci MA (2014). Pharmacological stimulation of sigma-1 receptors has neurorestorative effects in experimental parkinsonism. Brain : A Journal of Neurology..

[18] Rung JP, Rung E, Helgeson L, Johansson AM, Svensson K, Carlsson A (2008). Effects of (−)-OSU6162 and ACR16 on motor activity in rats, indicating a unique mechanism of dopaminergic stabilization. Journal of Neural Transmission..

[19] Ponten H, Kullingsjo J, Lagerkvist S, Martin P, Pettersson F, Sonesson C (2010). In vivo pharmacology of the dopaminergic stabilizer pridopidine. European Journal of Pharmacology..

[20] de Yebenes JG, Landwehrmeyer B, Squitieri F, Reilmann R, Rosser A, Barker RA (2011). Pridopidine for the treatment of motor function in patients with Huntington’s disease (MermaiHD): a phase 3, randomised, double-blind, placebo-controlled trial. The Lancet Neurology..

[21] Esmaeilzadeh M, Kullingsjo J, Ullman H, Varrone A, Tedroff J (2011). Regional cerebral glucose metabolism after pridopidine (ACR16) treatment in patients with Huntington disease. Clinical Neuropharmacology..

[22] Huntington Study Group HI (2013). A randomized, double-blind, placebo-controlled trial of pridopidine in Huntington's disease. Movement Disorders : Official Journal of the Movement Disorder Society..

[23] Lundin A, Dietrichs E, Haghighi S, Goller ML, Heiberg A, Loutfi G (2010). Efficacy and safety of the dopaminergic stabilizer pridopidine (ACR16) in patients with Huntington's disease. Clinical Neuropharmacology..

[24] Garcia-Miralles M, Geva M, Tan JY, Yusof N, Cha Y, Kusko R, et al. Early pridopidine treatment improves behavioral and transcriptional deficits in YAC128 Huntington disease mice. JCI Insight. 2017;2(23). 10.1172/jci.insight.95665 PMC5752291 29212949

[25] Sahlholm K, Arhem P, Fuxe K, Marcellino D (2013). The dopamine stabilizers ACR16 and (−)-OSU6162 display nanomolar affinities at the sigma-1 receptor. Molecular Psychiatry..

[26] Dyhring T, Nielsen EO, Sonesson C, Pettersson F, Karlsson J, Svensson P (2010). The dopaminergic stabilizers pridopidine (ACR16) and (−)-OSU6162 display dopamine D(2) receptor antagonism and fast receptor dissociation properties. European Journal of Pharmacology..

[27] Sahlholm K, Sijbesma JW, Maas B, Kwizera C, Marcellino D, Ramakrishnan NK (2015). Pridopidine selectively occupies sigma-1 rather than dopamine D2 receptors at behaviorally active doses. Psychopharmacology..

[28] Francardo V, Recchia A, Popovic N, Andersson D, Nissbrandt H, Cenci MA (2011). Impact of the lesion procedure on the profiles of motor impairment and molecular responsiveness to L-DOPA in the 6-hydroxydopamine mouse model of Parkinson's disease. Neurobiology of Disease..

[29] Lundblad M, Andersson M, Winkler C, Kirik D, Wierup N, Cenci MA (2002). Pharmacological validation of behavioural measures of akinesia and dyskinesia in a rat model of Parkinson's disease. The European Journal of Neuroscience..

[30] Blume SR, Cass DK, Tseng KY (2009). Stepping test in mice: a reliable approach in determining forelimb akinesia in MPTP-induced Parkinsonism. Experimental Neurology..

[31] Winkler C, Bentlage C, Nikkhah G, Samii M, Bjorklund A (1999). Intranigral transplants of GABA-rich striatal tissue induce behavioral recovery in the rat Parkinson model and promote the effects obtained by intrastriatal dopaminergic transplants. Experimental Neurology..

[32] Mysona BA, Zhao J, Smith S, Bollinger KE (2018). Relationship between sigma-1 receptor and BDNF in the visual system. Experimental Eye Research..

[33] West MJ (1999). Stereological methods for estimating the total number of neurons and synapses: issues of precision and bias. Trends in Neurosciences..

[34] Westin JE, Lindgren HS, Gardi J, Nyengaard JR, Brundin P, Mohapel P (2006). Endothelial proliferation and increased blood-brain barrier permeability in the basal ganglia in a rat model of 3,4-dihydroxyphenyl-L-alanine-induced dyskinesia. The Journal of Neuroscience : The Official Journal of the Society for Neuroscience..

[35] Song DD, Haber SN (2000). Striatal responses to partial dopaminergic lesion: evidence for compensatory sprouting. The Journal of Neuroscience : The Official Journal of the Society for Neuroscience..

[36] Cicchetti F, Brownell AL, Williams K, Chen YI, Livni E, Isacson O (2002). Neuroinflammation of the nigrostriatal pathway during progressive 6-OHDA dopamine degeneration in rats monitored by immunohistochemistry and PET imaging. The European Journal of Neuroscience..

[37] Perry VH, Teeling J (2013). Microglia and macrophages of the central nervous system: the contribution of microglia priming and systemic inflammation to chronic neurodegeneration. Seminars in Immunopathology..

[38] Paxinos G, Franklin KBJ (2001). The Mouse Brain in Stereotaxic Coordinates.

[39] Granado N, Ares-Santos S, Tizabi Y, Moratalla R. Striatal Reinnervation Process after Acute Methamphetamine-Induced Dopaminergic Degeneration in Mice. Neurotoxicity Research. 2018. 10.1007/s12640-018-9925-z 29934756

[40] Chang JW, Wachtel SR, Young D, Kang UJ (1999). Biochemical and anatomical characterization of forepaw adjusting steps in rat models of Parkinson's disease: studies on medial forebrain bundle and striatal lesions. Neuroscience..

[41] Cousins MS, Salamone JD (1996). Skilled motor deficits in rats induced by ventrolateral striatal dopamine depletions: behavioral and pharmacological characterization. Brain Research..

[42] Fujimoto M, Hayashi T, Urfer R, Mita S, Su TP (2012). Sigma-1 receptor chaperones regulate the secretion of brain-derived neurotrophic factor. Synapse..

[43] Garcia-Martinez JM, Perez-Navarro E, Gavalda N, Alberch J (2006). Glial cell line-derived neurotrophic factor promotes the arborization of cultured striatal neurons through the p42/p44 mitogen-activated protein kinase pathway. Journal of Neuroscience Research..

[44] Svensson KA, Falcone JF, Johansson AM, Perry KW, MJ F, editors. The actions of the dopamine stabilizer ACR16, but not (−)-OSU6162, in behavioral and neurochemical assays are not dependent on the presence of functional dopamine D2 receptors, 39th Annual Meeting, Society for Neuroscience; 2009; Chicago.

[45] Ryskamp D, Wu J, Geva M, Kusko R, Grossman I, Hayden M (2017). The sigma-1 receptor mediates the beneficial effects of pridopidine in a mouse model of Huntington disease. Neurobiology of Disease..

[46] Geva M, Kusko R, Soares H, Fowler KD, Birnberg T, Barash S (2016). Pridopidine activates neuroprotective pathways impaired in Huntington Disease. Human Molecular Genetics..

[47] Kordower JH, Olanow CW, Dodiya HB, Chu Y, Beach TG, Adler CH (2013). Disease duration and the integrity of the nigrostriatal system in Parkinson's disease. Brain : A Journal of Neurology..

[48] Lima FAV, Joventino IP, Joventino FP, de Almeida AC, Neves KRT, do Carmo MR (2017). Neuroprotective Activities of Spirulina platensis in the 6-OHDA Model of Parkinson's Disease Are Related to Its Anti-Inflammatory Effects. Neurochemical Research..

[49] Lima LAR, Lopes MJP, Costa RO, Lima FAV, Neves KRT, Calou IBF (2018). Vitamin D protects dopaminergic neurons against neuroinflammation and oxidative stress in hemiparkinsonian rats. Journal of Neuroinflammation..

[50] Tentillier N, Etzerodt A, Olesen MN, Rizalar FS, Jacobsen J, Bender D (2016). Anti-Inflammatory Modulation of Microglia via CD163-Targeted Glucocorticoids Protects Dopaminergic Neurons in the 6-OHDA Parkinson's Disease Model. The Journal of Neuroscience : The Official Journal of the Society for Neuroscience..

[51] Baluchnejadmojarad T, Mansouri M, Ghalami J, Mokhtari Z, Roghani M (2017). Sesamin imparts neuroprotection against intrastriatal 6-hydroxydopamine toxicity by inhibition of astroglial activation, apoptosis and oxidative stress. Biomedicine & Pharmacotherapy = Biomedecine & Pharmacotherapie..

[52] Lazzarini M, Martin S, Mitkovski M, Vozari RR, Stuhmer W, Bel ED (2013). Doxycycline restrains glia and confers neuroprotection in a 6-OHDA Parkinson model. Glia..

[53] Morroni F, Sita G, Tarozzi A, Cantelli-Forti G, Hrelia P (2014). Neuroprotection by 6-(methylsulfinyl)hexyl isothiocyanate in a 6-hydroxydopamine mouse model of Parkinsons disease. Brain research..

[54] Hong J, Sha S, Zhou L, Wang C, Yin J, Chen L (2015). Sigma-1 receptor deficiency reduces MPTP-induced parkinsonism and death of dopaminergic neurons. Cell death & Disease..

[55] Tronci E, Francardo V (2018). Animal models of L-DOPA-induced dyskinesia: the 6-OHDA-lesioned rat and mouse. Journal of Neural Transmission..

[56] Peviani M, Salvaneschi E, Bontempi L, Petese A, Manzo A, Rossi D (2014). Neuroprotective effects of the Sigma-1 receptor (S1R) agonist PRE-084, in a mouse model of motor neuron disease not linked to SOD1 mutation. Neurobiology of Disease..

[57] Maurice T, Su TP (2009). The pharmacology of sigma-1 receptors. Pharmacology & Therapeutics..

[58] Hayashi T (2003). Su TP. Intracellular dynamics of sigma-1 receptors (sigma(1) binding sites) in NG108-15 cells. The Journal of Pharmacology and Experimental Therapeutics.

[59] Hayashi T, Su TP (2003). Sigma-1 receptors (sigma(1) binding sites) form raft-like microdomains and target lipid droplets on the endoplasmic reticulum: roles in endoplasmic reticulum lipid compartmentalization and export. The Journal of Pharmacology and Experimental Therapeutics.

[60] Tillerson JL, Cohen AD, Philhower J, Miller GW, Zigmond MJ, Schallert T (2001). Forced limb-use effects on the behavioral and neurochemical effects of 6-hydroxydopamine. The Journal of Neuroscience : The Official Journal of the Society for Neuroscience..

[61] Robertson GS, Robertson HA. Evidence that the substantia nigra is a site of action for L-DOPA. Neuroscience Letters. 1988;89(2):204–8. 10.1016/0304-3940(88)90382-5 3393296

[62] Yurek DM, Hipkens SB (1993). Intranigral injections of SCH 23390 inhibit amphetamine-induced rotational behavior. Brain Research..

[63] McGarry A, Kieburtz K, Abler V, Grachev ID, Gandhi S, Auinger P (2017). Safety and Exploratory Efficacy at 36 Months in Open-HART, an Open-Label Extension Study of Pridopidine in Huntington's Disease. Journal of Huntington's Disease..

[64] Squitieri F, Di Pardo A, Favellato M, Amico E, Maglione V, Frati L (2015). Pridopidine, a dopamine stabilizer, improves motor performance and shows neuroprotective effects in Huntington disease R6/2 mouse model. Journal of Cellular and Molecular Medicine..

