# Supplementary material for: Pridopidine Induces Functional Neurorestoration Via the Sigma-1 Receptor in a Mouse Model of Parkinson’s Disease
Source: Neurotherapeutics. 2019 Feb 12;16(2):465–79. doi: 10.1007/s13311-018-00699-9 (PMC6554374; doi:10.1007/s13311-018-00699-9)
Supplement: Supplementary file 8 — (DOCX 21 kb) [file 13311_2018_699_MOESM8_ESM.docx]

**Supplementary Table S1.**

| **Plasma** | | | | | | | | |
| --- | --- | --- | --- | --- | --- | --- | --- | --- |
| **Dose, RoA** | | **T_max_ [h]** | **C_max_ [ng/mL]** | | **AUC_0-last_ [ng*h*kg/mL*mg]** | | **C_max_/dose [ng*kg/mL*mg]** | **AUC_0-last_ /dose[ng*h/mL]** |
| **30 po** | | 0.25 | 4699 | | 6839 | | 157 | 228 |
| **30 sc** | | 0.5 | 4007 | | 7566 | | 134 | 252 |
| **po/sc** | | NA | 117% | | 90% | | NA | NA |
| **Brain** | | | | | | | | |
| **Dose, RoA** | **T_max_ [h]** | | | **C_max_ [ng/mL]** | | **AUC_0-last_ [ng*h/mL]** | **C_max_/dose [ng*kg/mL*mg]** | **AUC_0-last_ /dose[ng*h/mL]** |
| **30 po** | 0.25 | | | 13196 | | 19028 | 440 | 634 |
| **30 sc** | 1 | | | 11555 | | 19774 | 385 | 659 |
| **po/sc** | NA | | | 88% | | 96% | NA | NA |

**Supplementary Table S1**. **Pridopidine plasma and brain exposure following oral (p.o) or subcutaneous (s.c) administration.** These data indicate that 7-days oral (p.o) pridopidine administration produces plasma and brain exposure similar to that observed after subcutaneous (s.c) administration. No pre-dose levels could be detected for any administration route either in plasma or in brain, suggesting that pridopidine does not accumulate. This result supports the assumption that the pharmacokinetics obtained at day 7 is indicative of the one at day 3. Data were collected from 3 mice per time-point per arm, treated with once daily dose of pridopidine 30 mg/kg (salt) through oral or s.c. administration. NA, not applicable.

**Supplementary Table S2**

| **Pridopidine concentration** | **% bound in mouse** |
| --- | --- |
| 0.1 µM (0.03µg/mL) | 78.3±4.74 |
| 1 µM (0.28 µg/mL) | 50.9±3.71 |
| 10 µM (2.8 µg/mL) | 62.4±3.50 |
| 50 µM (14 µg/mL) | 54.7±3.44 |
| 100 µM (28.14 µg/mL) | 50.9±10.5 |

**Suppl Table 2. Brain tissue homogenate binding of pridopidine in mice**. These data show which percentage of pridopidine remains bound to brain proteins at different concentrations. Note: %Bound = [(Residual concentration in brain homogenate - Concentration in PBS) at 3 hours/Total concentration in brain homogenate] * 100%.
